# The Role of Immunotherapy in Resectable Non-Small Cell Lung Cancer

**DOI:** 10.32604/or.2026.076281

**Published:** 2026-04-22

**Authors:** Francesco Petrella, Andrea Cara, Enrico Mario Cassina, Lidia Libretti, Emanuele Pirondini, Federico Raveglia, Maria Chiara Sibilia, Antonio Tuoro

**Affiliations:** 1 Department of Thoracic Surgery, Fondazione IRCCS San Gerardo dei Tintori, Monza, Italy; 2 Department of Oncology and Hemato-Oncology, University of Milan, Milan, Italy

**Keywords:** Non-small cell lung cancer, immunotherapy, lung resection, immune checkpoint inhibitors, pathological response

## Abstract

The advent of immune checkpoint inhibitors (ICIs) targeting PD-1, PD-L1, and CTLA-4 has transformed the therapeutic landscape of advanced non-small cell lung cancer (NSCLC), and recent clinical trials have extended their application to resectable disease. Multiple randomized phase III trials have demonstrated that neoadjuvant and adjuvant immunotherapy, particularly when combined with platinum-based chemotherapy, significantly improves pathological complete response (pCR), major pathological response (MPR), event-free survival (EFS), disease-free survival (DFS), and overall survival (OS) compared to chemotherapy alone. Several key questions remain unresolved—including whether preoperative or postoperative immunotherapy yields superior outcomes, whether adjuvant therapy provides additional benefit after neoadjuvant immune checkpoint inhibitors plus chemotherapy (ICI-CT), and how best to identify the patients most likely to benefit from each strategy. This review will critically examine the current evidence, clinical trial landscape, and future directions for immunotherapy in resectable NSCLC.

## 1 Introduction

Lung cancer remains the leading cause of cancer-related mortality worldwide, with NSCLC accounting for the majority of cases. Despite advances in surgical techniques and adjuvant chemotherapy, recurrence rates after resection for early-stage non-small cell lung cancer (NSCLC) remain substantial, underscoring the need for more effective perioperative strategies [1–3]. The advent of ICIs targeting Programmed Death-1 (PD-1), programmed death-ligand 1 (PD-L1), and Cytotoxic T-Lymphocyte-Associated protein 4 (CTLA-4) has transformed the therapeutic landscape of advanced NSCLC, and recent clinical trials have extended their application to resectable disease [4–6]. Multiple randomized phase III trials have demonstrated that neoadjuvant and adjuvant immunotherapy, particularly when combined with platinum-based chemotherapy, significantly improves pathological complete response (Pcr), major pathological response (MPR), event-free survival (EFS), disease-free survival (DFS), and overall survival (OS) compared to chemotherapy alone [7–12]. In particular, perioperative immunotherapy combined with chemotherapy significantly prolongs OS in resectable NSCLC compared to chemotherapy alone, with the greatest benefit observed in stage II–III disease and higher PD-L1 expression. Agents such as nivolumab, pembrolizumab, atezolizumab, and durvalumab have received Food and Drug Administration (FDA) approval for perioperative use in resectable NSCLC, reflecting their efficacy and manageable safety profiles [6–8]. Notably, neoadjuvant chemoimmunotherapy has achieved pCR rates up to 28% and has not increased perioperative morbidity, supporting its integration into standard care. Despite these advances, several challenges persist, including optimal patient selection, timing and duration of immunotherapy, and the role of biomarkers such as PD-L1 expression in guiding therapy [1–4]. Ongoing research aims to refine these strategies and address unresolved questions regarding the synergism between chemotherapy and immunotherapy, as well as the necessity of adjuvant ICI following neoadjuvant treatment [10–12]. This review will critically examine the current evidence, clinical trial landscape, and future directions for immunotherapy in resectable NSCLC.

## 2 Adjuvant Approach

For resectable NSCLC, the standard therapeutic strategy has traditionally consisted of surgical resection followed by adjuvant platinum-based doublet chemotherapy in patients considered at high risk for recurrence. Current National Comprehensive Cancer Network (NCCN) guidelines generally do not recommend adjuvant chemotherapy for stage IA disease [13]. In contrast, for stage IB tumors, adjuvant platinum-based therapy may be considered when high-risk pathological features are present, although consensus remains incomplete [14]. Evidence indicates that adjuvant platinum chemotherapy improves 5-year OS by approximately 5.4% compared with surgery alone [15]. The rationale for using adjuvant immunotherapy in patients with resected NSCLC is based on the significant improvement in DFS compared to observation or placebo after standard adjuvant chemotherapy. In fact, it has been demonstrated that adjuvant immunotherapy enhances antitumor immune surveillance and reduces recurrence risk after surgery and chemotherapy, establishing it as a new standard for eligible patients with resected early-stage NSCLC [16]. Combining immunotherapy with chemotherapy can yield synergistic effects, as cytotoxic agents promote tumor cell lysis and neoantigen exposure, enhancing immune recognition [7]. Currently, two ICIs have regulatory approval in the adjuvant setting for resectable NSCLC. Atezolizumab is indicated following platinum-based chemotherapy for completely resected stage II–IIIA tumours with PD-L1 tumour proportion score (TPS) ≥1% (≥50% in some regions), while pembrolizumab is approved for stage IB–IIIA disease regardless of PD-L1 status.

The IMpower010 trial evaluated adjuvant atezolizumab after resection and platinum chemotherapy in stage IB–IIIA NSCLC [17]. Among 1005 randomized patients, atezolizumab significantly improved DFS in stage II–IIIA cases with PD-L1 ≥1%, particularly those with PD-L1 ≥50%, while results in the overall intent-to-treat (ITT) population did not reach statistical significance. The exploratory OS benefit observed in the trial among patients with resected NSCLC whose tumours had PD-L1 expression of 50% or greater was a HR for OS of 0.43 (95% CI 0.24–0.78), favouring adjuvant atezolizumab over best supportive care after platinum-based chemotherapy in the stage II–IIIA population. This indicates a substantial reduction in the risk of death in this subgroup, with the benefit not seen in patients with lower PD-L1 expression levels. DFS benefit was greatest in patients with tumors ≥5 cm or nodal involvement. In the trial, subgroup analyses demonstrated that patients belonging to stage II or higher experienced a more pronounced reduction in the risk of recurrence or death with atezolizumab compared to best supportive care after adjuvant platinum-based chemotherapy. These findings demonstrate that the magnitude of benefit from adjuvant atezolizumab is greater in patients with stage II or higher disease, particularly those with PD-L1–positive tumors, compared to the overall population that includes stage IB disease. The HR for DFS in the stage II–IIIA population (which includes tumors ≥5 cm and/or node-positive disease) was 0.79 (95% CI 0.64–0.96), indicating a substantial benefit [17]. These findings led to FDA approval of adjuvant atezolizumab for resected stage II–IIIA NSCLC with PD-L1 ≥1% [18,19]. Conversely, the PEARLS/KEYNOTE-091 trial demonstrated that adjuvant pembrolizumab significantly prolonged DFS in tumors ≥4 cm or nodal metastases, independent of nodal status or PD-L1 expression [20]. In fact, pembrolizumab significantly improved DFS in the overall population, which included all patients with tumors ≥4 cm or nodal metastases. The median DFS was 53.6 months with pembrolizumab vs. 42.0 months with placebo (HR 0.76, 95% CI 0.63–0.91; *p* = 0.0014), demonstrating a clinically meaningful benefit. This DFS benefit was consistent across subgroups, including those defined by tumor size, and specifically applies to patients with tumors ≥4 cm or nodal metastases, as this was the minimum size for stage IB inclusion [20].

In contrast, durvalumab did not show a DFS advantage in the adjuvant setting. The Canadian Cancer Trials Group (CCTG BR.31) evaluated adjuvant durvalumab (20 mg/kg IV every 4 weeks for 12 cycles) vs. placebo in patients with completely resected stage IB (≥4 cm) to IIIA non-small cell lung cancer (NSCLC), with or without adjuvant chemotherapy. The primary analysis focused on patients with EGFR-/ALK-tumors and PD-L1 tumor cell expression ≥25%. After a median follow-up of 60 months, adjuvant durvalumab did not improve DFS compared to placebo (stratified HR 0.93, 95% CI 0.71–1.25; *p* = 0.64), and no benefit was seen in secondary subgroups either. Grade 3–4 adverse events were more frequent with durvalumab (26% vs. 20%).

Plausible reasons for this negative outcome, especially when compared to positive adjuvant immunotherapy trials (e.g., IMpower010, KEYNOTE-091), include differences in surgical quality (such as completeness of nodal dissection), patient selection (e.g., inclusion of lower-risk stage IB patients, variable PD-L1 expression thresholds), and trial design (e.g., optional adjuvant chemotherapy, stratification factors, and timing of immunotherapy initiation). The BR.31 trial specifically stratified by extent of nodal dissection, but heterogeneity in surgical practices across centers may have influenced recurrence rates and diluted the effect of adjuvant therapy. Additionally, the inclusion of patients with lower PD-L1 expression and optional chemotherapy may have contributed to the lack of observed benefit [21].

Together, these findings suggest that the benefits of adjuvant immunotherapy in resectable NSCLC are influenced by tumor stage, PD-L1 expression, and prior treatment context (in particular: whether patients received adjuvant platinum-based chemotherapy after surgery before starting immunotherapy, as well as the timing and completeness of surgical resection), underscoring the need for personalized, biomarker-driven treatment strategies (Table 1). The benefit of adjuvant immunotherapy appears to be agent-specific and context-dependent. IMpower010 (atezolizumab) showed a strong PD-L1-dependent effect, while KEYNOTE-091 (pembrolizumab) demonstrated a broader DFS benefit across PD-L1 subgroups, possibly due to differences in trial design and patient selection.

**Table 1:** Major studies involving immunotherapy in the adjuvant setting in resectable non-small cell lung cancer (NSCLC).

Study	Regimen	Population	Comparator	Key Endpoints	Main Results (If Available)	Sample Size	Follow-Up (Duration)	Primary Endpoint Met	Refer ences
IMpower010	Atezolizumab	Resected stage IB (≥4 cm)-IIIA NSCLC, post-platinum chemo, EGFR/ALK wild-type, PD-L1 stratified	Best supportive care	DFS (primary), OS (secondary)	DFS benefit in stage II-IIIA, especially PD-L1 ≥1% (HR 0.66); OS trend in PD-L1 ≥50%	1005	32.2 months	**Yes**	[17]
PEARLS/ KEYNOTE-091	Pembrolizumab	Resected stage IB (≥4 cm)-IIIA NSCLC, post-platinum chemo (14% no chemo), all-comers (PD-L1/EGFR/ALK not restricted)	Placebo	DFS (primary), OS (secondary)	DFS benefit (HR 0.76); benefit seen regardless of PD-L1 or EGFR status; no OS benefit yet	1177	34.3 months	**Yes**	[20]
ANVIL (NCT02595944 ALCHEMIST)	Nivolumab	Resected stage IB-IIIA NSCLC, EGFR/ALK wild-type, post-standard adjuvant therapy	Observation	DFS, OS	Ongoing; no published results yet	~900	Ongoing	**Pending**	NA
BR31 (NCT02273375 CCTG)	Durvalumab	Resected stage IB (≥4 cm)-IIIA NSCLC, post-platinum chemo	Placebo	DFS, OS	Ongoing; no published results yet	1380	~36 months	**Pending**	NA

Note: DFS, disease-free survival; OS, overall survival; HR, hazard ratio; NA, not applicable.

## 3 Neoadjuvant Approach

The introduction of immunotherapy has prompted extensive investigation of ICIs as neoadjuvant treatments, either alone or in combination with chemotherapy and radiotherapy. This strategy is based on the rationale that an intact tumor immune microenvironment may enhance responsiveness to ICIs [22]. Several studies have explored different therapeutic combinations: for instance, PD-1/PD-L1 inhibitors paired with CTLA-4 blockade in the NEOSTAR trial, the integration of ICIs with stereotactic body radiation therapy as demonstrated by Altorki et al. [23], and the inclusion of novel agents in regimens such as those tested in the NEOCOAST trial [24]. Across these studies, adverse event profiles remained consistent with known ICI toxicities, with grade 3–5 events occurring in approximately 10%–30% of patients. Neoadjuvant monotherapy with anti–PD-(L)1 agents has produced MPR rates ranging from 6.7% to 45%. The randomized phase II NEOSTAR trial evaluated nivolumab alone vs. nivolumab combined with ipilimumab in patients with resectable NSCLC. Combination therapy achieved an MPR of 38% among 21 patients, with many also reaching a pCR. Building on these results, NEOSTAR transitioned into a platform trial structure, consisting of sequential, single-arm phase II studies to further test the impact of ICI-based regimens. Reported MPR rates were 50% (11/22) and 32.1% (7/22) in subsequent cohorts, confirming the potential of neoadjuvant immunotherapy [25]. By pairing the cytotoxic properties of chemotherapy with the immune activation induced by ICIs, combination regimens aim to achieve a synergistic effect that could prolong survival and improve overall outcomes. In 2022, the FDA approved the combination of neoadjuvant nivolumab and platinum-doublet chemotherapy for resectable NSCLC, based on the favorable results of the phase III CheckMate 816 trial [26]. Following this, the NCCN recommended the same regimen for patients with stage IB–IIIA or select stage IIIB (T3N2) disease. CheckMate 816, a randomized phase III open-label trial, enrolled patients with resectable stage IB (≥4 cm) to IIIA NSCLC (AJCC v7), Eastern Cooperative Oncology Group (ECOG) performance status ≤1, and no EGFR or ALK alterations. Participants received either nivolumab plus platinum-based chemotherapy or chemotherapy alone, both given every three weeks for three cycles before surgery. The addition of nivolumab significantly improved EFS (EFS: 31.6 vs. 20.8 months) and MPR (24.0% vs. 2.2%). Importantly, it remains the only neoadjuvant-only phase III trial demonstrating a statistically and clinically significant OS advantage at five years, with OS rates of 65% for the combination vs. 55% for chemotherapy alone. Patients who achieved a pCR derived particularly strong benefit, with a 5-year OS of 95% compared to 56% in non-pCR cases [27] (Table 2).

**Table 2:** Major studies involving immunotherapy in the neoadjuvant setting in resectable non-small cell lung cancer (NSCLC).

Study	Regimen	Population	Major Pathologic Response (MPR)	Pathologic Complete Response (pCR)	Key Efficacy/Safety Findings	Sample Size	Follow-Up (Duration)	Primary Endpoint Met	References
CheckMate 816	Nivolumab + platinum chemo (3 cycles)	IB-IIIA	36.9%	24.0%	Improved pCR, EFS, OS vs. chemo; no ↑ AEs	358	41.3 months	**Yes**	[27]
NEOSTAR	Nivolumab (3 cycles) vs. Nivo + Ipilimumab (3 cycles)	I-IIIA	21.7% (Nivo), 38.1% (Nivo + Ipi)	8.7% (Nivo), 28.6% (Nivo + Ipi)	Combo superior to mono; no ↑ surgical AEs	44	24 months	**Yes**	[25]
Pilot study	Nivolumab (2 cycles)	I-IIIA	45%	10%	Feasible, safe, promising MPR	NA	NA	NA	[8]
PRINCEPS	Atezolizumab (1 cycle)	IB-IIIA	0%	0%	No MPR/pCR; safe	30	12 months	**No**	[8]
IONESCO	Durvalumab (3 cycles)	IB-IIIA	18%	7%	Early closure due to post-op mortality	46	12 months	**No**	[8]
Sintilimab study	Sintilimab (2 cycles)	IA-IIIB	40.5%	Not reported	Promising MPR, safe	**40**	**61 months**	**Yes**	[8]

Note: EFS, event-free survival; OS, overall survival; AEs, adverse events.

## 4 Perioperative Approach

The integration of immunotherapy in the perioperative setting—encompassing both neoadjuvant and adjuvant phases—has emerged as a key strategy in the treatment of resectable NSCLC. Although combining treatments before and after surgery complicates the ability to isolate the effects of each phase, this approach may offer meaningful advantages, particularly for patients who show suboptimal response to neoadjuvant therapy.

The NADIM trial, a multicenter phase 2 study, explored a perioperative regimen consisting of three cycles of neoadjuvant paclitaxel–carboplatin plus nivolumab followed by surgery and one year of adjuvant nivolumab in stage IIIA NSCLC. Each cycle consisted of paclitaxel (200 mg/m^2^) and carboplatin (AUC 6 mg/mL per min) given intravenously every 21 days, in combination with nivolumab (360 mg), prior to surgical resection. At 5-year follow-up, PFS reached 65.0% (95% CI 49.4%–76.9%), while OS was 69.3% (95% CI 53.7%–80.6%). Disease progression occurred in 24% of participants, and 30% died, mostly due to relapse. Grade ≥3 treatment-related toxicities occurred in 30% of patients during the neoadjuvant phase and 19% during the adjuvant phase, with no delays to surgery or unexpected long-term safety concerns [28].

NADIM II compared neoadjuvant platinum doublet chemotherapy (PDC) with or without nivolumab, followed by adjuvant nivolumab, in stage IIIA–IIIB resectable NSCLC. The number of neoadjuvant chemotherapy cycles administered was three cycles of paclitaxel–carboplatin plus nivolumab, given every 21 days prior to surgery. The planned duration of adjuvant nivolumab therapy was six months, administered at a dose of 480 mg intravenously every four weeks following R0 resection. The trial demonstrated a striking improvement in pCR with the perioperative nivolumab regimen (37% vs. 7%; RR 5.34, *p* = 0.002) [29].

The phase III AEGEAN trial assessed durvalumab added to neoadjuvant PDC followed by adjuvant durvalumab, vs. PDC plus placebo. The number of neoadjuvant chemotherapy cycles administered was four cycles of platinum-based chemotherapy, given every three weeks in combination with durvalumab prior to surgery. The planned duration of adjuvant durvalumab therapy was 12 cycles, administered every four weeks after surgery, for up to one year. With a median follow-up of 11.7 months and exclusion of EGFR/ALK-positive tumors, median EFS was not reached in the durvalumab group compared with 24.9 months in the control arm. Durvalumab reduced the risk of progression, recurrence, or death by 32% (HR 0.68) and increased pCR (17.2% vs. 4.3%; *p* < 0.001). Despite these improvements, the incremental contribution of adjuvant therapy remains uncertain, as overall outcomes mirrored those seen with purely neoadjuvant chemoimmunotherapy in CheckMate 816 [30].

CheckMate 77T, a randomized phase III, double-blind study, tested neoadjuvant nivolumab plus chemotherapy followed by adjuvant nivolumab, *vs*. matched placebo regimens. The nivolumab arm exhibited a superior 18-month EFS rate (70.2% vs. 50.0%) and a higher pCR rate (25.3% vs. 4.7%) [31]. In the RATIONALE-315 phase III trial conducted in China, adding tislelizumab to neoadjuvant and adjuvant chemotherapy in stage II–IIIA resectable NSCLC markedly improved outcomes. The tislelizumab arm achieved higher pCR (40.7% vs. 5.7%) and MPR rates (56.2% vs. 15.0%), independent of PD-L1 expression or histology. Definitive surgery was performed in 84.1% of patients receiving tislelizumab and 76.2% of those on placebo. Safety findings were consistent with established chemoimmunotherapy profiles [32].

The NEOToRCH trial was a randomized, double-blind, placebo-controlled phase III study evaluating perioperative toripalimab plus platinum-based chemotherapy vs. chemotherapy alone in patients with resectable stage II or III NSCLC without EGFR or ALK alterations. Patients received three cycles of neoadjuvant toripalimab or placebo with chemotherapy, followed by surgery, one cycle of adjuvant toripalimab or placebo with chemotherapy, and then up to 13 cycles of maintenance toripalimab or placebo monotherapy.

The main findings were a significant improvement in EFS and MPR rates in the toripalimab arm. In the interim analysis of 404 patients with stage III NSCLC, the median EFS was not reached in the toripalimab group *vs*. 15.1 months in the placebo group (HR 0.40, 95% CI 0.28–0.57; *p* < 0.001). The 2-year EFS rate was 66.7% with toripalimab vs. 46.1% with placebo. MPR was 48.5% vs. 8.4%, and pathological complete response (pCR) was 24.8% vs. 1.0% in favour of toripalimab. The benefit was consistent across PD-L1 subgroups, with the greatest EFS improvement in patients with PD-L1 ≥1%. Safety was manageable; immune-related adverse events were more frequent with toripalimab, but grade ≥3 adverse events, fatal events, and treatment discontinuations were similar between groups [33].

Finally, the KEYNOTE-671 trial was a global, randomized, double-blind, placebo-controlled phase 3 study evaluating perioperative immunotherapy in resectable stage II, IIIA, or IIIB (N2) NSCLC. Patients (N = 797) were randomized 1:1 to receive four cycles of neoadjuvant pembrolizumab (200 mg IV every 3 weeks) plus cisplatin-based chemotherapy, followed by surgery and up to 13 cycles of adjuvant pembrolizumab (200 mg IV every 3 weeks), or matched placebo plus chemotherapy and surgery, then adjuvant placebo. Randomization was stratified by stage, PD-L1 expression, histology, and region. The main efficacy outcomes were dual primary endpoints of EFS and OS. At a median follow-up of 36.6 months, perioperative pembrolizumab significantly improved EFS (median 47.2 vs. 18.3 months; HR 0.59, 95% CI 0.48–0.72) and OS (36-month OS: 71% vs. 64%; HR 0.72, 95% CI 0.56–0.93) compared to placebo. Pembrolizumab also increased MPR (30.2% vs. 11.0%) and pCR (18.1% vs. 4.0%) rates. These benefits were consistent across key subgroups, including PD-L1 expression and disease stage. Safety outcomes showed higher rates of grade 3–5 treatment-related adverse events with pembrolizumab (45% vs. 38%), but rates of treatment-related death were low and similar (1% in both arms). The most common adverse events were cytopenia, nausea, and fatigue, consistent with chemotherapy. Immune-related adverse events were more frequent with pembrolizumab but manageable. Importantly, perioperative pembrolizumab did not adversely affect surgical feasibility, R0 resection rates, or quality of life [34] (Table 3).

**Table 3:** Major studies involving immunotherapy in the peri-operative setting in resectable non-small cell lung cancer (NSCLC).

Study	Regimen	Population	Comparator	Primary Endpoint	Key Efficacy Results	Sample Size	Follow-Up (Duration)	Primary Endpoint Met	Refer ences
NADIM	Nivolumab + paclitaxel/carboplatin (neoadj) → adjuvant nivolumab	Resectable IIIA NSCLC	None (single-arm)	24-mo PFS	5-yr OS: 69.3%; 5-yr PFS: 65%	46	58.7 months	Yes	[28]
NADIM II	Nivolumab + chemo (perioperative)	Resectable IIIA NSCLC	Chemo alone	PFS, OS, pCR	Improved pCR, EFS, OS vs. chemo alone	86	26.7 months	Yes	[29]
AEGEAN	Durvalumab + platinum chemo (neoadj) → adjuvant durvalumab	Resectable II–IIIB NSCLC	Chemo + placebo	EFS, pCR	EFS HR: 0.68; pCR improved	802	25.4 months	Yes	[30]
CheckMate 77T	Nivolumab + platinum chemo (neoadj) → adjuvant nivolumab	Resectable II–IIIB NSCLC	Chemo + placebo	EFS, pCR	EFS HR: ~0.68; pCR improved	461	25.4 months	Yes	[31]
RATIONALE-315	Tislelizumab + platinum chemo (neoadj) → adjuvant tislelizumab	Resectable II–IIIA NSCLC	Chemo + placebo	MPR, EFS	EFS HR: 0.56; MPR: 56% vs. 15%	388	18.0 months	Yes	[32]
Neotorch	Toripalimab + platinum chemo (neoadj) → adjuvant toripalimab	Resectable II–IIIB NSCLC	Chemo + placebo	EFS, pCR	EFS HR: ~0.58; pCR improved	404	18.0 months	Yes	[33]
KEYNOTE-671	Pembrolizumab + platinum chemo (neoadj) → adjuvant pembrolizumab	Resectable II–IIIB NSCLC	Chemo + placebo	OS, EFS	OS HR: 0.72; EFS HR: 0.59; pCR improved	797	25.2 months	Yes	[34]

Note: OS, overall survival; PFS, progression-free survival; EFS, event-free survival; HR, hazard ratio; MPR, major pathological response; pCR, pathological complete response.

## 5 Biomarkers

The most important biomarkers for guiding perioperative immunotherapy in resectable NSCLC are programmed death-ligand 1 (PD-L1), minimal residual disease (MRD), tumor mutational burden (TMB), and microsatellite instability (MSI).

PD-L1 expression is the most established biomarker. Higher PD-L1 levels (≥1%, and especially ≥50%) are associated with greater pathological response rates, event-free survival, and overall survival in perioperative immunotherapy trials, but benefit is observed across all PD-L1 subgroups; thus, lack of PD-L1 expression should not exclude patients from immunotherapy. PD-L1 is routinely assessed by immunohistochemistry.

Minimal residual disease (MRD), typically measured by circulating tumor DNA (ctDNA), is emerging as a powerful prognostic biomarker. Postoperative ctDNA positivity strongly predicts relapse risk and may help stratify patients for adjuvant therapy, although its predictive value for immunotherapy response is not yet validated for treatment selection. MRD-negative patients have lower recurrence risk, but current assays have limitations in sensitivity and clinical utility.

Tumor mutational burden (TMB) is associated with increased response rates and survival with immunotherapy, as high TMB reflects greater neoantigen load and immunogenicity. However, TMB is not routinely used in clinical practice due to technical challenges and a lack of standardized cutoffs, though it may provide additional predictive information beyond PD-L1.

Microsatellite instability (MSI) and mismatch repair deficiency are rare in NSCLC (<1%), but when present, they confer high TMB and increased likelihood of response to immune checkpoint inhibitors. MSI status is best assessed by next-generation sequencing and immunohistochemistry, and may guide immunotherapy in select cases [33–35].

In summary, PD-L1 is the primary biomarker for perioperative immunotherapy selection, MRD is promising for relapse risk stratification, TMB may add predictive value, and MSI identifies a rare subset highly responsive to immunotherapy.

While PD-L1 is a validated biomarker for perioperative immunotherapy selection, MRD, TMB, and MSI are exploratory biomarkers in resectable NSCLC. None have been prospectively validated in randomized trials to guide perioperative treatment decisions. These biomarkers should not currently be used in routine clinical practice to determine treatment selection, to avoid inappropriate extrapolation.

## 6 Discussion

Preoperative attrition—defined as the proportion of patients who ultimately do not undergo the planned lung cancer resection—is a critical parameter in evaluating neoadjuvant and perioperative immunochemotherapy (ICI-CT) strategies [35]. Across the trials analyzed, attrition rates before surgery ranged from 16% to 22% in ICI-CT arms and 19% to 27% in chemotherapy-only arms, values broadly consistent with the historical 4%–18% attrition observed in neoadjuvant chemotherapy trials. Although adverse events (AEs) or disease progression can prevent surgery, both were relatively infrequent in studies involving ICI-CT. AEs accounted for only 1%–6% of attrition in ICI-CT arms (vs. 0%–4% with chemotherapy alone), whereas disease progression caused attrition in 3%–7% of ICI-CT patients (vs. 10%–15% under chemotherapy alone). Other potentially avoidable reasons—including patient refusal, loss of fitness for surgery, or surgical cancellation—occurred in 7%–12% of ICI-CT patients and 8%–15% of those receiving chemotherapy alone [28–34].

Before initiating systemic therapy, it is essential to determine whether both the primary tumour and mediastinal lymph nodes are technically resectable. Completeness of resection remains a critical surgical outcome: the International Association for the Study of Lung Cancer (IASLC) R-classification defines R0 resection as no residual tumor, negative surgical margins, adequate nodal assessment (≥6 nodal stations, including subcarinal and ≥2 mediastinal stations), and the highest nodal station negative. Importantly, negative pleural lavage cytology is not required [36]. In the neoadjuvant and perioperative ICI-CT studies, R0 resection rates were consistently high; in adjuvant trials, they were uniformly 100% because R0 status was required for enrollment. Among neoadjuvant/perioperative studies, CheckMate 816 reported the lowest R0 rates, with substantial geographic variability (65% in North America vs. 90% in Europe and 90% in Asia), possibly reflecting differences in application of the International Association for the Study of Lung Cancer (IASLC) criteria, patient selection, or attempts to convert borderline-resectable tumors into operable ones [27]. Most operations across trials consisted of lobectomy or pneumonectomy. In CheckMate 816, pneumonectomy occurred less frequently after neoadjuvant ICI-CT (11%–17%) compared with chemotherapy alone (21%–25%). By contrast, perioperative trials showed no meaningful difference between the two treatment arms (ICI-CT 7%–11% vs. chemotherapy 8%–14%) [27]. Although AEGEAN excluded patients anticipated to need pneumonectomy, postoperative pneumonectomy rates remained similar between arms (7% vs. 8%) [30].

Patients with resectable NSCLC—defined as clinical stage IB to IIIA and select IIIB disease—are appropriate candidates for neoadjuvant immunotherapy when they do not harbor actionable driver mutations. Major phase III trials, in fact, have demonstrated that neoadjuvant chemoimmunotherapy in patients without driver mutations leads to significantly improved pCR, MPR, and event-free survival compared to chemotherapy alone, without increasing perioperative morbidity.

Patients with EGFR, ALK, ROS1, RET, ERBB2, or NTRK mutations should be excluded from neoadjuvant immunotherapy due to minimal efficacy and increased risk of toxicity. For these patients, alternative strategies are recommended: the FDA has approved adjuvant osimertinib for patients with resected NSCLC harboring EGFR exon 19 deletions or exon 21 L858R mutations. The optimal management for these patients is complete surgical resection followed by adjuvant osimertinib. For less common EGFR mutations, the benefit of adjuvant (tyrosine kinase inhibitors) TKIs is less clear, and management should be individualized, ideally within a multidisciplinary framework.

Different neoadjuvant immunotherapy studies in resectable NSCLC show markedly variable rates of pCR due to heterogeneity in PD-L1 expression, chemotherapy backbone, number of cycles administered, and patient selection. PD-L1 expression is a key factor: tumors with higher PD-L1 expression (≥50%) are significantly more likely to achieve pCR, as demonstrated in both meta-analyses and real-world cohorts. The chemotherapy backbone also influences pCR rates. Platinum-doublet regimens combined with PD-1/PD-L1 inhibitors consistently yield higher pCR rates than monotherapy, but the specific agents (e.g., cisplatin vs. carboplatin, pemetrexed vs. paclitaxel) and their immunomodulatory effects may contribute to variability. Patient heterogeneity—including stage, nodal status, comorbidities, and molecular profile—further explains discrepancies. Advanced nodal disease (N2) is associated with lower pCR rates, and older age may reduce the likelihood of proceeding to surgery and achieving pCR.

Recent network meta-analyses and indirect comparisons indicate that neoadjuvant immunotherapy may be preferable to adjuvant immunotherapy, as it elicits a more robust systemic antitumor response and avoids the increased toxicity and financial burden of prolonged adjuvant therapy, without a clear survival advantage for the latter.

Treatment-related AEs with neoadjuvant ICI-CT were comparable to chemotherapy-alone regimens. Nevertheless, ICIs introduce additional immune-related toxicities with variable onset and duration. Across phase 3 trials, safety remained acceptable, discontinuations were infrequent, and perioperative mortality mirrored the control groups [25,27–34]. The lower incidence of grade ≥3 AEs reported in adjuvant trials (22%–34%) likely reflects the sequential administration of chemotherapy followed by ICI, in contrast to the concurrent ICI-CT used in neoadjuvant and perioperative studies, where grade ≥3 AE rates reached 41%–70%. PD-L1 tumour expression continues to serve as a key biomarker predicting benefit from ICI. In CheckMate 816, EFS improved progressively with increasing PD-L1 expression: 0.85 (PD-L1 <1%), 0.58 (1%–49%), and 0.24 (≥50%) [32–34]. Perioperative trials showed less uniform trends, with EFS hazard ratios ranging from 0.65–0.80 (PD-L1 <1%), 0.31–0.76 (1%–49%), and 0.26–0.71 (≥50%). A meta-analysis of 43 neoadjuvant trials enrolling over 5400 resectable NSCLC patients found superiority of ICI-CT over chemotherapy alone across surgical, pathological, and efficacy outcomes [37]. Patients with PD-L1 <1% still experienced EFS improvement, though without an OS advantage; greater PD-L1 expression correlated with larger EFS benefits. Interpretation of these results is complicated by the inclusion of perioperative studies, where adjuvant therapy may influence EFS and OS [37]. In the adjuvant setting, IMpower010 demonstrated increasing DFS and OS benefit with higher PD-L1 expression [17]. The 5-year OS hazard ratios for stage II–IIIA disease were 0.47 for PD-L1 ≥50% and 0.77 for PD-L1 ≥1% [17].

While some phase 2 neoadjuvant ICI studies reported similar long-term outcomes irrespective of PD-L1 status [38], PD-L1 currently remains the most clinically relevant biomarker of ICI responsiveness. Decisions regarding pre- vs. postoperative therapy require thorough assessment of both medical operability and surgical resectability, ideally before systemic treatment begins. Medical operability depends on cardiopulmonary reserve and comorbidities, whereas resectability is determined by radiologic and staging features of the tumor and lymph nodes. These decisions should be made within a multidisciplinary tumor board, which has been shown to improve staging accuracy, adherence to guidelines, and overall treatment outcomes [39–42].

Early multidisciplinary evaluation, appropriate patient selection, and efficient management of treatment-related adverse events are key strategies to improve surgery rates and optimize outcomes for patients receiving perioperative immunotherapy for resectable NSCLC. These approaches, supported by recent clinical trial data and consensus guidelines, are essential for integrating perioperative immunotherapy into routine practice while minimizing risks and maximizing benefit.

Neoadjuvant chemoimmunotherapy is associated with higher rates of grade 3 or higher adverse events, primarily due to chemotherapy, while adjuvant immune checkpoint inhibitor therapy alone generally has lower toxicity and does not negatively impact quality of life (QoL).

QoL declines during the neoadjuvant phase, likely due to chemotherapy and tumor-related symptoms, but returns to baseline during the adjuvant phase, regardless of whether patients receive active immunotherapy or placebo. This pattern is consistent across perioperative trials and is supported by patient-reported outcomes.

The rationale for choosing neoadjuvant or perioperative immunotherapy over upfront surgery alone is to enhance long-term survival and reduce recurrence by leveraging immune priming, pathological response assessment, and eradication of micrometastatic disease, with a favorable safety profile. Anyway, further data on optimal patient selection and long-term outcomes are still evolving.

The unplanned analyses from the CheckMate 816 and CheckMate 77T trials presented at the World Conference on Lung Cancer (WCLC) 2024 focused on the relationship between pathological response, EFS, and overall survival OS in the context of perioperative immunotherapy for resectable NSCLC. Limitations of these unplanned analyses include their exploratory nature, lack of statistical power, absence of pre-specified hypotheses, and potential for selection bias. The inability to directly compare neoadjuvant vs. adjuvant components, short follow-up duration, and underrepresentation of certain demographic groups (e.g., Black patients) further restrict generalizability. However, these unplanned analyses highlight the potential prognostic value of pCR and ctDNA clearance and suggest benefit from perioperative immunotherapy, but definitive conclusions regarding optimal sequencing and the independent effect of adjuvant therapy cannot be drawn due to methodological limitations. Combined exploratory analyses from these trials suggest that patients who do not achieve pCR after neoadjuvant therapy may derive additional benefit from adjuvant immunotherapy, whereas those with pCR have excellent outcomes with surveillance alone. Adjuvant immunotherapy should be considered for patients without pCR after neoadjuvant chemoimmunotherapy and surgery, particularly those with residual disease, high-risk pathological features, or incomplete ctDNA clearance.

Long-term management of immune-related adverse events (irAEs) in perioperative immunotherapy for resectable NSCLC requires ongoing surveillance and multidisciplinary care. Chronic irAEs such as hypothyroidism, adrenal insufficiency, diabetes mellitus, and inflammatory arthropathies may persist beyond immunotherapy completion and often necessitate lifelong hormone replacement, immunosuppression, or specialist follow-up. The American Society of Clinical Oncology recommends regular monitoring for late endocrine, rheumatologic, and neurologic toxicities, with prompt referral to subspecialists and tailored survivorship care plans to address sequelae and optimize quality of life [43].

Persistent irAEs can impact physical and psychosocial functioning, and management may include long-term corticosteroids, hormone replacement, and rehabilitation. Surveillance for delayed toxicities is essential, as some may emerge months to years after therapy [43].

Mechanisms of resistance to immune checkpoint inhibitors in this setting are multifactorial. Primary resistance is often driven by tumor-intrinsic factors such as low PD-L1 expression, low tumour mutational burden, and mutations in STK11, KEAP1, or other genes that create an immunologically “cold” tumour microenvironment. Acquired resistance may develop through up-regulation of alternative immune checkpoints, loss of neoantigen expression, or changes in the tumour microenvironment that promote immune evasion, such as increased regulatory T cells or myeloid-derived suppressor cells. Both primary and acquired resistance limit the durability of response and are active areas of research, with combination strategies (e.g., dual checkpoint blockade, targeting co-inhibitory signals, or modulating the microenvironment) under investigation to overcome these barriers [44].

## 7 Conclusions

Neoadjuvant, perioperative, and adjuvant ICI-CT regimens are all validated, effective strategies that improve DFS and EFS relative to chemotherapy alone, with acceptable safety profiles. Peri-operative chemo-immunotherapy significantly improves OS in patients with resectable non-small cell lung cancer (NSCLC) compared to chemotherapy alone. These benefits are observed across disease stages (IB–IIIA), PD-L1 expression levels, and nodal involvement, and are accompanied by manageable toxicity profiles. The clinical implication is that perioperative chemo-immunotherapy is now considered a standard of care for eligible patients with resectable NSCLC, offering a substantial improvement in long-term survival outcomes over previous approaches.

Each approach is now an established standard of care in resectable NSCLC. Several key questions remain unresolved—including whether preoperative or postoperative immunotherapy yields superior outcomes, whether adjuvant therapy provides additional benefit after neoadjuvant ICI-CT, and how best to identify the patients most likely to benefit from each strategy. Choosing between pre- and postoperative approaches requires balancing the risk of preoperative surgical attrition, postoperative failure to receive systemic therapy, and the anticipated toxicities. Neoadjuvant therapy should be preferred over a postoperative approach in resectable NSCLC when maximizing the likelihood of pathologic complete response and major pathologic response is a priority, particularly in patients with stage IIA and higher disease, or in those with clinically node-negative T2b/T3 N0 tumors.

According to the IASLC consensus recommendations [45], several key principles have to be emphasized:

(1) A multidisciplinary team approach is critical for the evaluation and management of resectable NSCLC, ensuring individualized care and optimal treatment sequencing.
(2) Comprehensive biomarker testing (including EGFR, ALK, and PD-L1) is recommended for all patients with resectable NSCLC to guide therapy selection.
(3) For stage III resectable NSCLC without EGFR or ALK alterations, the preferred first-line approach is neoadjuvant platinum-based chemotherapy combined with immunotherapy, regardless of PD-L1 status.
(4) Neoadjuvant chemotherapy alone is reserved for those with contraindications to immunotherapy.
(5) For patients with sensitizing EGFR mutations or ALK rearrangements, adjuvant targeted therapy (e.g., osimertinib for EGFR, alectinib for ALK) is strongly preferred after resection, independent of adjuvant chemotherapy use.
(6) For resected stage II–IIIA NSCLC patients without EGFR or ALK alterations who did not receive neoadjuvant immunotherapy, adjuvant immunotherapy (atezolizumab or pembrolizumab) is recommended for patients with PD-L1 ≥50% and can be considered for patients with PD-L1 1%–49% following adjuvant chemotherapy.

These recommendations are designed to be practical and sensitive to global differences in resources and biology, and will evolve as new evidence emerges. The IASLC panel highlights the need for ongoing multidisciplinary collaboration and individualized care in the absence of definitive data for certain subgroups [45].

## Data Availability

Available upon request.

## Abbreviation

AEs: Adverse events
ALK: Anaplastic lymphoma kinase
BSC: Best supportive care
CI: Confidence interval
CTLA-4: Cytotoxic T-lymphocyte-associated protein 4
DFS: Disease-free survival
ECOG: Eastern cooperative oncology group
EGFR: Epidermal growth factor receptor
EFS: Event-free survival
FDA: Food and drug administration
HR: Hazard ratio
IASLC: International association for the study of lung cancer
ICIs: Immune checkpoint inhibitors
ICI-CT: Immune checkpoint inhibitors plus chemotherapy
irAEs: Immune-related adverse events
ITT: Intention-to-treat
MPR: Major pathological response
MRD: Minimal residual disease
MSI: Microsatellite instability
NCCN: National comprehensive cancer network
NSCLC: Non-small cell lung cancer
OR: Odds ratio
OS: Overall survival
pCR: Pathological complete response
PDC: Platinum doublet chemotherapy
PD-1: Programmed death-1
PD-L1: Programmed death-ligand 1
RMST: Restricted mean survival time
TMB: Tumor mutational burden
TKI: Tyrosine kinase inhibitor
TPS: Tumor proportion score
